# Alterations in N-glycosylation of HCV E2 Protein in Children Patients with IFN-RBV Therapy Failure

**DOI:** 10.3390/pathogens13030256

**Published:** 2024-03-15

**Authors:** Karolina Zimmer, Alicja M. Chmielewska, Paulina Jackowiak, Marek Figlerowicz, Krystyna Bienkowska-Szewczyk

**Affiliations:** 1 Laboratory of Virus Molecular Biology, Intercollegiate Faculty of Biotechnology of UG and MUG, University of Gdansk, Abrahama 58, 80-307 Gdansk, Poland; karolina.zimm@gmail.com (K.Z.); alicja.chmielewska@biotech.ug.edu.pl (A.M.C.); 2 Department of Biochemistry and Molecular Biology, Faculty of Health Sciences, University of Bielsko-Biala, 43-309 Bielsko-Biala, Poland; 3 Institute of Bioorganic Chemistry, Polish Academy of Sciences, 61-704 Poznań, Polandmarek.figlerowicz@ibch.poznan.pl (M.F.)

**Keywords:** hepatitis C virus, glycoproteins, glycosylation, neutralization antibodies, response to therapy

## Abstract

The glycosylation of viral envelope proteins plays an important role in virus biology and the immune response of the host to infection. Hepatitis C virus (HCV) envelope proteins E1 and E2, key players in virus entry and spread, are highly N-glycosylated and possess 4 (5 in certain genotypes) to 11 conserved glycosylation sites, respectively. Many published results based on recombinant proteins indicate that the glycan shield can mask the epitopes targeted by neutralizing antibodies. Glycan shifting within the conserved linear E2 region (412–423) could be one of the escape strategies used by HCV. In the present report, we isolated E2 genes from samples (collected before the IFN-RBV therapy) originating from pediatric patients infected with HCV gt 1a. We analyzed the biochemical properties of cloned E2 glycoprotein variants and investigated their glycosylation status. The sequencing of E2 genes isolated from patients who did not respond to therapy revealed mutations at N-glycosylation sites, thus leading to a lower molecular weight and a low affinity to both linear and conformational neutralizing antibodies. The loss of the glycosylation site within the conserved epitope (amino acid 417) impaired the binding with AP33, an antibody that potently neutralizes all genotypes of HCV. Our findings, based on clinical samples, confirm the influence of N-glycosylation aberrations on the antigenic and conformational properties of HCV E1/E2, which may possibly correlate with the outcome of therapy in patients.

## 1. Introduction

Chronic Hepatitis C virus (HCV) infection affects 58 million people worldwide and constitutes a serious global health problem [1,2]. In the past, the standard treatment against HCV involved therapy with ribavirin and pegylated interferon. In recent years, a repertoire of new direct-acting antiviral drugs (DAAs) has been introduced to the market, increasing the rate of HCV clearance to over 90%. However, the limited availability and high cost of DAA treatments, together with the lack of an effective vaccine, are the major obstacles affecting the successful control of the disease burden [3]. Moreover, it is estimated that 1.5 million of new HCV infections occur globally each year. There remain numerous gaps in the understanding of the immune response against HCV, which, together with the high genetic variability of the virus, hamper the development of a vaccine.

E1 and E2 are type I transmembrane proteins that are embedded in the virus envelope. As functional heterodimers, they interact with the host cellular receptors to mediate the entry of the virus into the host cell. Being located at the virion surface, they are exposed to the host immune system and are the main target of humoral immune responses, including neutralizing antibodies. The ectodomains of both E1 and E2 are heavily glycosylated, with glycans representing one-third of the E1E2 molecular mass. E1 harbors four N-linked glycans at amino acid positions 196, 209, 234, and 305 of genotype 1a (strain H77). A fifth glycosylation site can be found in genotypes 1b and 6 at position 250, or in genotype 2b at position 299 [4]. E2 possesses up to 11 N-linked glycosylation sites. Despite the genetic diversity of E2, most glycosylation sites are highly conserved. The important role played by N-glycosylation in the escape of viruses from neutralizing antibodies is supported by the appearance of escape mutants in vitro [5] and in patient samples [6]. Importantly, the glycosylation pattern on E1E2 might be crucial for the effective induction of neutralizing immune responses by candidate subunit vaccines [7,8,9].

Here, we analyzed the E2 sequences from HCV genotype 1a in samples that were collected from a group of 10 pediatric patients in Poland before the onset of therapy. The patients were subsequently treated with the standard of care at the time, which was combined ribavirin–interferon, and were divided into three groups: three responders (R), six non-responders (NR), and a single transient responder (TR). Surprisingly, we observed mutations leading to the loss of N-glycosylation sites in all subsequent NR patients but not in the TR patients and R patients. A further biochemical analysis of the cloned and expressed mutated E2 proteins showed differences in CD81 receptor binding and in the recognition of E2 proteins by a panel of conformational antibodies. For the first time, our study shows the possible correlation between N-glycosylation aberrations, together with observed antigenic and conformational E1/E2 changes, and the outcome of therapy in patients.

## 2. Materials and Methods

### 2.1. Patients’ Samples

The HCV samples were collected in 2005 from 10 pediatric patients with chronic hepatitis caused by HCV-1a infection. All children were hospitalized and in the pre-treatment period before the initiation of interferon–ribavirin therapy, which was the only available treatment at this time [10]. The samples were primarily used for the studies of HCV genetic variability and analysis of quasispecies diversification in regard to the E2 gene as described in Figlerowicz et al. [11] and Jackowiak et al. [12]. HCV RNA was isolated from the patients’ sera and E2 sequences were amplified by RT-PCR. The method used to generate samples was described previously [11]. From these stored cloned samples, we obtained fragments containing a 1166 nt portion of the E1E2 coding sequence, which were used in this study. For further analysis the samples were cloned and sequenced and, according to the information obtained from the former clinical studies, divided into three therapy–response profiles: three sustained responders (SR), one transient responder (TR), and six non-responders (NR).

### 2.2. Antibodies

The mouse anti-E2 monoclonal antibodies (MAbs) AP33 and ALP98 have been described previously [13,14] and were a kind gift from A.H. Patel. The polyclonal anti-E2 antibody (PAbs) was purchased from Biorad. The anti-E1 MAb was purchased from Santa Cruz Biotechnology. The human anti-E2 mAbs CBH-4B, HC-1, and HC-11 [15,16] were a kind gift from S. K. Foung. The mouse anti-E2 Mab HC84.1 [17] was a kind gift from A.H. Patel.

### 2.3. Cell Lines

Human epithelial kidney (HEK) 293T cells were grown in Dulbecco’s modified eagle medium (DMEM, Sigma Aldrich, St. Louis, MO, USA) supplemented with 10% fetal bovine serum (FBS) and penicillin–streptomycin solution. Cells were maintained at 37 °C in a humidified 5% CO_2_ atmosphere. HEK 293T cells originated from the collection of the Laboratory of Virus Molecular Biology

### 2.4. Construction of Chimeric E1E2 Expression Plasmids

The plasmid encoding HCV gt 1a strain H77c E1E2 (GenBank accession number AF011751) was previously described [18]. Due to the lack of amplified original patient-derived E1 sequences, chimeric E1E2 heterodimers were constructed using an H77c laboratory strain-derived E1 and patient-derived sequences from E2. The cloning strategy was based on a three-step assembly PCR, due to the characteristic sequence of each patient-derived E2 variant. In the first step, H77c was used as a template and the following primers were employed:

E1_FOR: 5′-GTCCCTTGCGTTCGCGAGGGTAACGC-3′ and E1_REV: 5′-CCTTCGCCCAGTTCCCCACCATGGAG-3′; a ~400 bp product was amplified. Next, using an appropriate set of primers and the patient-derived E1/E2 sequence, a 1100 bp product was obtained; this covered the whole E2 coding sequence and a small, conserved terminal fragment of the E1 coding sequence. Finally, both fragments were assembled into a 1400 bp product, using primers E1_FOR and the appropriate E2_REV. After digestion with the Bsp68I (Fermentas) and NotI (Fermentas) restriction enzymes, the final PCR product was cloned into the previously digested H77c_pcDNA3.1 expression vector. All obtained chimeric constructs were verified via DNA sequencing and analysis in Geneious^®^ software (version 2019.1.2, Biomatters, Inc., Boston, MA, USA). To confirm the E1 and E2 expression, 293T cells were transfected with the constructed E1E2 plasmids using the JetPRIME reagent (Polyplus transfection). Then, 48 h after transfection, the cells were lysed using a buffer containing 20 mM of Tris-HCl, 1 mM of EDTA, 150 mM of NaCl, and 0.5% Triton X-100. E1E2 expression was detected via the immunostaining and immunoprecipitation of cells; this was followed by Western blotting.

### 2.5. In Situ Immunostaining of Cells

E1E2-transfected 293T cell monolayers were washed with PBS, frozen at −70 °C for 15 min, and fixed with 4% paraformaldehyde. The cells were probed with either anti-E2 mAb ALP98 or anti-E1 mAb diluted in PBS with 5% fetal bovine serum (FBS), 0.1% sodium azide, and 1% Tween-80. The secondary HRP-conjugated anti-mouse antibody (Santa Cruz) was diluted at 1:2000 in PBS with 5% FBS and 1% Tween-80. The reaction was developed using the Nova-RED substrate kit (Vector Laboratories, Mowry Ave Newark, CA, USA).

### 2.6. E1E2 Immunoprecipitation

293T cell lysates (prepared as described above) containing E2 variants were incubated overnight with goat anti-E2 PAbs (Biorad, Hercules, CA, USA) and then incubated for 16 h with Protein A/G resin (Thermo Scientific, Waltham, MA, USA). Antibody–glycoprotein complexes were eluted from the resin and separated on 12% SDS-PAGE; this was followed by detection via Western blotting.

### 2.7. GNA-Capture ELISA

First, 96-well Microlon ELISA-Plates (Greiner) were coated with a 10 µg/mL solution of Galanthus nivalis lectin (Sigma Aldrich, St. Louis, MO, USA) and used to capture recombinant glycoproteins from the lysates of 293T cells transiently transfected with wild-type or chimeric E1E2-coding plasmids. The lysate obtained from mock-transfected cells served as a negative control. Bound glycoproteins were detected with the appropriate primary antibodies, followed by anti-species IgG–HRP conjugate (Santa Cruz Biotechnology, Dallas, TX, USA) and the 1-Step Turbo TMB-ELISA substrate (Thermo Scientific Waltham, MA, USA).

### 2.8. SDS-PAGE and Western Blotting Analysis

Analysis of the E2 glycoprotein molecular mass and antibody recognition was carried out via SDS-PAGE using 10% polyacrylamide gels. After electrophoresis, the proteins were transferred onto a polyvinylidene difluoride (PVDF, Millipore) membrane via semi-dry electrotransfer; subsequently, the membranes were blocked overnight at 4 °C with 3% non-fat milk in TBST (Tris-buffered saline/0.1% Tween 20). After blocking, the membranes were incubated for 1 h at RT with the appropriate primary antibody solution, washed with TBST, and incubated with horseradish peroxidase conjugate (HRP) (Santa Cruz Biotechnology, Dallas, TX, USA). The results were developed using the substrate for enhanced chemiluminescence (Thermo Scientific, Waltham, MA, USA)

### 2.9. Glycosylation Status Analysis

The N-glycosylation status of the E2 variants was analyzed using Net-N-Glyc 1.0 software (Department of Health Technology Ørsteds Plads, Lyngby, Denmark, http://www.cbs.dtu.dk/services/NetNGlyc/ accessed on 12 March 2018).

### 2.10. CD81-LEL-GST Fusion Protein Production

For the purification of recombinant large extracellular loop (LEL) of the human CD81 receptor fused to Glutathione S-transferase (GST), the BL21 competent cells were transformed with plasmids that code GST-CD81 LEL. The cell pellets were then lysed, and the lysates were applied to Glutathione Sepharose resin (Sigma Aldrich, St. Louis, MO, USA) and incubated overnight at 4 °C. The CD81-LEL-GST fusion protein was eluted with elution buffer (1 M Tris-HCl, pH 8.0/0.3% glutathione), and then fractions of the eluate were collected and their protein content analyzed via SDS-polyacrylamide gel electrophoresis (SDS-PAGE) and Coomassie Brilliant Blue R-250 staining. The peak fractions were pooled, aliquoted, and stored at −80 °C.

### 2.11. CD81-Binding Assay

First, 96-well Microlon ELISA-Plates (Greiner, Greiner Bio-One, Kremsmünster, Austria) were coated with 5 µg/well of recombinant GST-hCD81-LEL for 16 h at 4 °C. After incubation, the plates were washed with PBS/0.05% Tween 20 and unspecific binding sites were blocked via incubation for 3 h with 250 µL/well of blocking buffer (3% BSA/PBS/0.05% Tween 20). Then, 293T lysates containing an equal amount of chimeric E1E2 heterodimer variants were added and incubated overnight at 4 °C. Mock-transfected cell lysates served as a negative control. The next day, the plates were extensively washed with washing buffer and 100 µL/well of mouse anti-E2 primary antibody in 3% BSA/PBS/0.05% Tween 20 (1:500) was added. After 3 h of incubation at RT, the plates were washed and incubated for 1 h with 100 µL/well of the appropriate horseradish peroxidase conjugate in 3% BSA/PBS/0.05% Tween 20 (1:500). The plates were washed as described above and the reaction was developed by adding 100 µL/well of 1-Step Turbo TMB-ELISA substrate (Thermo Scientific, Waltham, MA, USA).

### 2.12. ELISA with Conformation-Sensitive Antibodies

First, 96-well Microlon ELISA-Plates (Greiner) were coated with HEK293 lysates containing an equal amount of chimeric E1E2 heterodimer variants and incubated overnight at 4 °C. Mock-transfected cell lysates served as a negative control. The next day, the plates were blocked via incubation for 3 h with 250 µL/well of blocking buffer (3% BSA/PBS/0.05% Tween 20). After washing, 100 µL/well of the conformational-sensitive anti-E2 primary antibody diluted in 3% BSA/PBS/0.05% Tween 20 was added. All antibodies (HC-1, HC-11, HC-84.1, CBH-4B) were used at a final concentration of 10 mg/mL. The ALP98 antibody served as a control. After 1 h of incubation at RT, the plates were washed and incubated for 1 h with 100 µL/well of the appropriate horseradish peroxidase conjugate in 3% BSA/PBS/0.05% Tween 20. The plates were washed as described above and the reaction was developed by adding 100 µL/well of 1-Step Turbo TMB-ELISA substrate (Thermo Scientific, Waltham, MA, USA).

### 2.13. Statistics

Data were analyzed and visualized using GraphPad Prism 8.0.2 (GraphPad Software, La Jolla, CA, USA). The significance comparisons were calculated using a two-tailed Student’s *t*-test.

## 3. Results

### 3.1. Collection and Analysis of Patient Samples

The HCV samples were collected in 2005 from pediatric patients with chronic hepatitis caused by HCV infection. The ten pediatric patients (six females and four males) aged 8–16 years with chronic hepatitis C (all infected with gt 1a) were subjected to combined interferon–ribavirin therapy in 2005 [10]. After 72 weeks of treatment and follow-up, the patients’ response to the conducted therapy was evaluated (Table 1). From the patients’ sera, HCV RNA was isolated and stored as RT-PCR products.

This study included ten archival PCR product samples originating from the patients (collected at time point T0) that contained a 1166 nt fragment of the HCV E1/E2 coding sequence [11]. Due to the fact that the patients presented three different therapy–response profiles ([11] and Table 1), we decided to test the hypothesis that there is a possible correlation between the genetic diversity of the HCV E2 glycoprotein and the therapy outcome. At this time, the original patient-derived E1 glycoprotein sequences were not available and, in the absence of E1, the E2 glycoprotein remained ineffective. Therefore, to study the properties of E2 in the context of the functional E1E2 heterodimer, we constructed a panel of chimeric sequences; this consisted of the E1 sequence (H77c isolate, genotype 1a) followed by the in-frame inserted sequence of a patient-derived E2. Due to the large number of quasispecies, the most frequent E2 variants were chosen for the reconstruction of all heterodimers.

### 3.2. E1E2 Heterodimer Reconstruction

**Figure 1:**
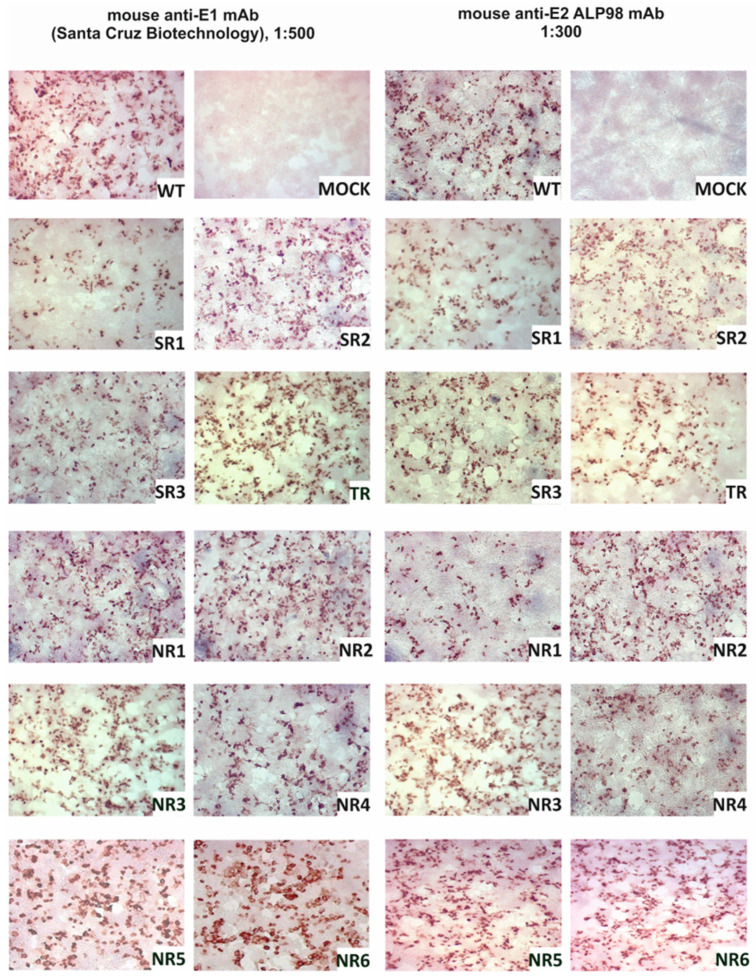
Confirmation of E1E2 expression in HEK293 cells. Detection of E1 and E2 glycoproteins via immunohistochemical staining in fixed cells. Then, 48 h post-transfection with the plasmids carrying sequences coding for reconstructed E1E2 heterodimers, the cells were fixed and probed with anti-E1 mAb or anti-E2 Alp98 mAbs. Mock-transfected HEK cells were used as a negative control. NR—no response, SR—sustained response, TR—transient response.

The E1E2 glycoprotein was transiently expressed in HEK293 cells and the expression was confirmed via immunoprecipitation and in situ immunostaining. The immunohistochemical staining of HEK293 cells after transfection was performed in parallel with immunoprecipitation in order to visualize the expression of the E1E2 proteins directly in cells. All reconstructed heterodimers were recognized by both the anti-E1 mAb and the anti-E2 ALP98 mAb (Figure 1).

**Figure 2:**
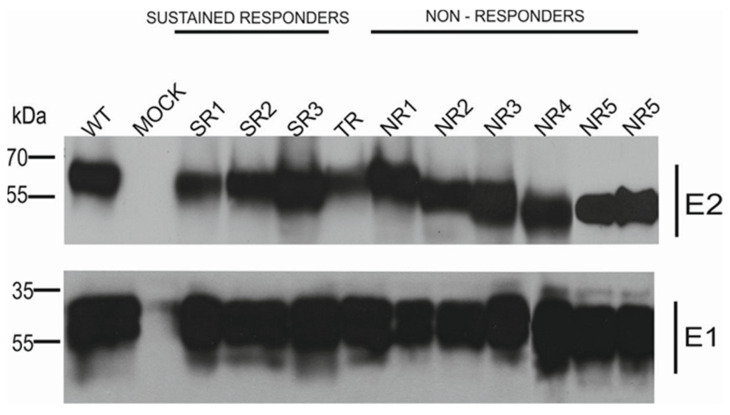
Analysis of immunoprecipitated E1E2 heterodimers. After the transfection of HEK293 with plasmids expressing either WT (H77c) or chimeric E1E2 heterodimers, immunoprecipitation was performed with whole-cell lysates and commercial anti-E2 pAbs. Western blotting of HCV glycoproteins was performed with both anti-E1 mAbs and anti-E2 ALP98 mAbs. Mock-transfected cells served as a negative control. NR—no response, SR—sustained response, TR—transient response.

The results of the immunoprecipitation and Western blotting analysis revealed a variability in the molecular weight of the E2 glycoprotein variants. Surprisingly, almost all E2 variants that originated from the NR group showed a decreased molecular weight, while the E2 bands representing samples from other patients were of the expected molecular weight, namely 62 kDa. The only exception was E1E2 heterodimer isolated from patient NR1 which shows a similar molecular weight to responders. As expected, the E1 glycoprotein exhibited a molecular weight of ~35 kDa, which was detected as multiple bands representing different glycoforms of protein (Figure 2).

### 3.3. E2 Glycoprotein Variants N-glycosylation Status Analysis

**Figure 3:**
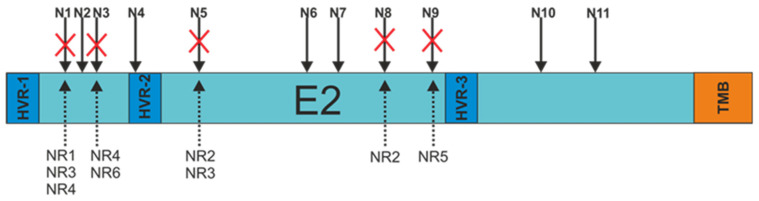
Schematic representation of the N-glycosylation sites present at the E2 surface. Red crosses indicate alterations in amino acid sequences resulting in the absence of putative N-linked glycan. N1-11—N-glycosylation sites, HVR-1,2,3—hypervariable regions, TMB—transmembrane domain, NR-1,2,3,4,5—E2 samples from non-responding patients.

In order to search for a probable explanation for the decrease in the molecular weight of the patient-derived E2 glycoproteins, each of the E2 variants was sequenced. The obtained sequences were used to analyze the N-glycosylation status using Net-N-Glyc 1.0 software. The research results revealed that there were alterations in the N-glycosylation patterns of the E2 sequences obtained from non-responders only. Each NR-derived E2 variant, even sample NR1 presenting unchanged molecular weight, lacked at least one N-glycosylation site (three samples with a single mutation and three samples with double mutations) (Figure 3).

The most frequent mutations involved the N1 (three samples), N3 (two samples), and N5 (two samples) glycosylation sites. The details of all changes in the E2 variant sequences that led to the loss of N-glycans are listed in Table 2.

**Figure 4:**
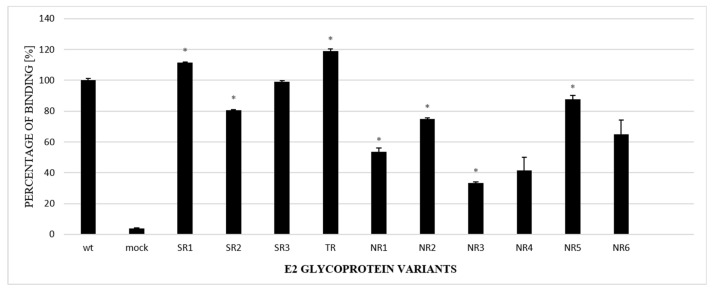
Analysis of the N-glycosylation status of E2 variants via lectin-capture ELISA. Lectin-bound E1E2 heterodimers from HEK293 cell lysates, transiently transfected with either WT or chimeric E1E2 heterodimers, were probed with ALP98 mAb. The binding of each sample is presented as a percentage of the signal for WT. Consistent data were obtained in three independent experiments. * *p* < 0.01.

The next steps included an analysis of the properties of the E2 variants. First, to further confirm the alterations in glycosylation, GNA-capture ELISA was used to test the ability of chimeric E1E2 to bind to lectins; this assay is commonly used for analyzing the attachment of viruses to glycans. The assay, performed with the lysates of HEK293 cells transiently transfected with either WT or chimeric E1E2, showed that all the E2 samples derived from non-responders had a lower affinity to lectins in comparison to the wild-type protein (Figure 4).

### 3.4. Functional Analysis of E1E2 Heterodimers

**Figure 5:**
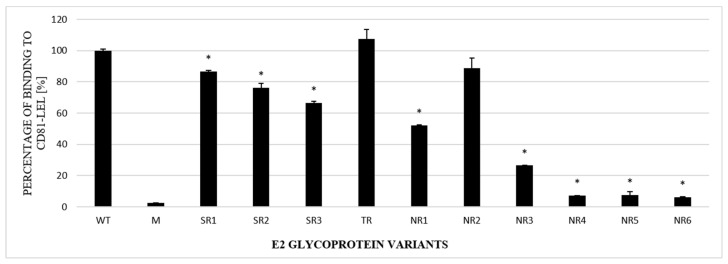
Efficacy of E2 binding to CD81-LEL. Immobilized CD81-LEL was used to capture the WT and chimeric E1E2 glycoprotein heterodimers from the lysates of transiently transfected HEK293 cells. CD81–E2 complexes were detected by ALP98 mAb. The binding of each sample is presented as a percentage of the signal for WT. Consistent data were obtained in three independent experiments. * *p* < 0.01.

The next step in the functional analysis of E1E2 heterodimers was the study of their affinity to the recombinant large extracellular loop (LEL) of the human CD81 receptor fused to Glutathione S-transferase (GST). Tetraspanin CD81 is one of the main HCV entry receptors and a key player in the HCV lifecycle [19]. In this experiment, ELISA plates were coated with recombinant purified GST-CD81-LEL and incubated with the lysates of cells expressing E1E2 proteins. Mock-transfected cell lysates were used as a negative control. The obtained results showed that the E1E2 heterodimers were able to bind recombinant CD81-LEL with variable efficiency. Most of the E2 samples derived from the NR group were bound with a significantly lower strength than WT and the samples originating from the SR or TR groups (Figure 5).

**Figure 6:**
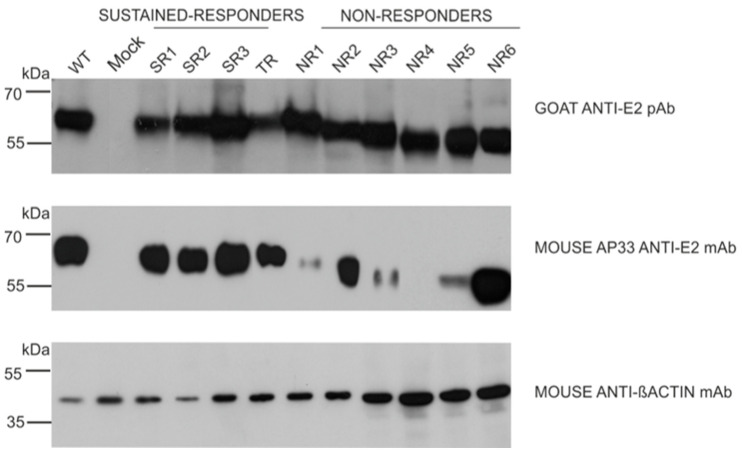
Detection of E2 glycoprotein variants via the neutralization of AP33 antibody. The E2 glycoproteins expressed in HEK293 were detected with anti-E2 pAbs (upper panel, control) or neutralizing anti-E2 AP33 mAb (middle panel) via Western blotting 48 h post-transfection. Gt1a H77c laboratory-strain-derived E1E2 proteins served as a positive control, mock-transfected cells were used as a negative control, and β-actin detection was performed as a sample loading control.

In the next step, we examined the ability of the panel of neutralizing and conformation-sensitive antibodies to recognize E2 variants. In the first experiments, we used the monoclonal antibody AP33; this antibody has the capacity to neutralize the most conserved epitope, encompassing residues 412 to 423 of E2, and was shown to recognize E2 from all HCV genotypes [13]. The results showed that the E2 variants carrying mutations in the N1 glycosylation site were less efficiently recognized than other samples, while the removal of N3 only did not affect AP33 binding. Moreover, the E2 glycoprotein lacking N1 and N3 glycosylation sites (sample NR4) was not detected by the AP33 antibody at all (Figure 6).

**Figure 7:**
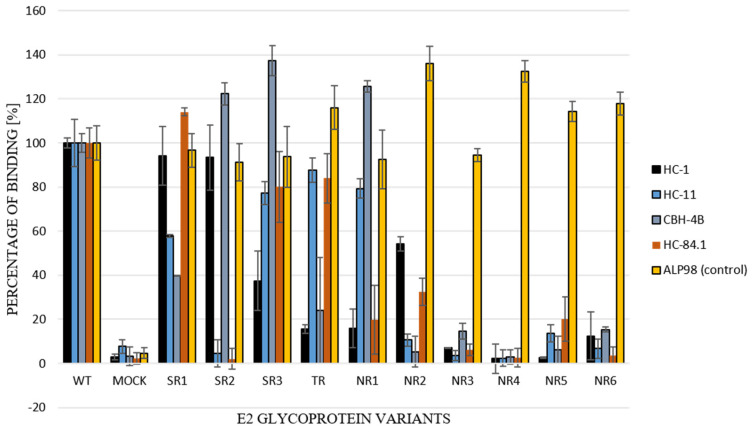
Recognition of E1E2 heterodimers by conformation-sensitive antibodies. E1E2 heterodimers (chimeric and wild type) from transiently transfected 293T cell lysates were probed with a panel of neutralizing and conformation-sensitive anti-E2 antibodies. The ALP98 Mab-recognizing linear epitope was used as a control. The binding of each sample is presented as a percentage of the signal for WT. Consistent data were obtained in three independent experiments.

For the further functional analysis of E2 glycoprotein variants, the impact of alterations in the E2 glycosylation pattern on local protein conformation and, therefore, on the recognition of HCV glycoprotein, was assessed via the performance of an ELISA assay with conformation-sensitive neutralizing antibodies. Patient-derived E2 glycoproteins were probed with a panel of four conformational-sensitive antibodies (HC-1, HC-11, CBH-4B, HC84.1) that recognized four out of the five antigenic domains localized within the E2 sequence [14,15,16,17]. The ALP98 monoclonal antibody-recognizing linear epitope was used as a control. The obtained results showed that the E2 samples were derived exclusively from non-responding patients and, because they lacked some glycans, were less efficiently recognized by all tested antibodies (Figure 7); this indicated the possible altered local conformation of the E2 glycoprotein.

## 4. Discussion

Although the development of directly acting antivirals (DAAs) has enabled HCV clearance in 90% of cases, their high cost limits the accessibility of these therapies in many countries. For this reason, the design of a preventive vaccine that induces the production of neutralizing antibodies is much needed. However, there are still some gaps in the understanding of the immune response against HCV, which, together with the high genetic variability of HCV, hampers the development of a vaccine.

HCV is characterized by high genetic polymorphism. The error-prone replication of viral RNA causes the accumulation of mutations and, in consequence, HCV does not form a homogenous population but exists as a pool of related but slightly divergent variants, which are called quasispecies. Genotype 1 is the most prevalent, comprising 46% of all infections [20]. The most variable component of the HCV particle is the envelope glycoprotein E2. The ectodomains of both E1 and E2 are heavily glycosylated, with E1 possessing up to 5 and E2 possessing up to 11 N-linked glycosylation sites. Nine glycosylation sites are conserved across HCV genotypes, and they are located at positions 417 (E2N1), 423 (E2N2), 430 (E2N3), 448 (E2N4), 532 (E2N6), 556 (E2N8), 576 (E2N9), 623 (E2N10), and 645 (E2N11) in the H77 reference strain. N-glycosylation occurs on the asparagine residue present in asparagine–X–serine/threonine motifs, with X standing for any residue; this is with the exception of proline. The N-glycosylation sites in E1/E2 have been confirmed to be occupied by glycans [21].

In this report, we analyzed the sequence and functionality of E2 glycoproteins in samples derived from ten pediatric patients infected with HCV genotype 1a. The samples were collected before IFN-RBV treatment and, following the treatment, patients were grouped into three therapy–response profiles: responders, non-responders, and single transient responders. In order to recreate a functional heterodimer, the sequences of patient-derived E2 were fused to the E1 gene from the laboratory strain (H77c). We found that all HCV E2 sequences isolated from the NR patients were characterized by mutations that led to the loss of N-glycosylation sites.

It has been previously reported that only correctly folded E1 and E2 are able to form a non-covalent heterodimer [22,23]. In order to confirm the heterodimerization process of the reference gt1a E1 and patient-derived E2 expressed in mammalian cells, the immunoprecipitation assay was performed. Anti-E2 polyclonal antibodies were used to capture E1E2 complexes from the HEK293 cell lysates. The results showed similar levels of precipitated glycoproteins for each constructed chimeric heterodimer, which indicated that all the E2 proteins transiently expressed in HEK293 were able to form a heterodimer with E1. However, we detected differences in the molecular weight of the E2 glycoprotein. Almost all E2 proteins derived from the NR patients had a lower molecular weight than the E2 samples obtained from the TR and SR patients, which could be attributed to the loss of particular glycan moieties. The only exception was E1E2 heterodimer isolated from patient NR1 which shows a similar molecular weight to responders (Figure 2).

On the basis of these data, we decided to check the glycosylation status of the patient-derived E2 glycoprotein variants. An analysis of the patients’ E2 sequences with Net-N-Glyc 1.0 software (Department of Health Technology Ørsteds Plads, Lyngby, Denmark). Available online at http://www.cbs.dtu.dk/services/NetNGlyc/ (accessed on 12 March 2018) revealed mutations in the consensus asparagine–X serine/threonine motifs, leading to the loss of at least one N-glycosylation site for each of the non-responding patients. In addition, we analyzed the N-glycosylation of 100 randomly selected HCV 1a patient-derived E2 sequences that were available in the euHCVdb database. We found 18 sequences that showed the absence of glycosylation sites, suggesting that this type of mutation is not unique to the cohort tested in this study. The most frequent mutations occurred at positions N4, N5 and N6. A published analysis performed for E2 sequences obtained from gt 3a [5] showed that N-glycosylation site deletions were rare for N4 and N5, which suggests that the appearance of N-glycans mutations might be genotype-specific.

Glycan shielding has been suggested to be a masking mechanism of broadly neutralizing epitopes on HCV viral glycoproteins. AP33 is one of the most potent neutralizing antibodies and targets the highly conserved E2 epitope 412–423, which contains two N-glycosylation sites (417 E2N1, 423 E2N2) [24]. Our research revealed that all of the E2 variants possessing mutations at the N1 glycosylation site were less efficiently recognized by AP33 nAb than non-mutated variants. Moreover, the E2 glycoprotein lacking N1 and N3 glycosylation sites (sample NR4) was undetected by this antibody. In previous studies, mutations in E2 affecting the glycosylation status of the protein led to the escape of the virus from broadly neutralizing antibodies. Dhillon and co-workers [25] showed that cell culture HCV isolates (HCVcc) carrying mutations at N415 and N417 (E2N1) were resistant to the AP33 antibody. Pantua and others [5] also reported that the E2N1 HCVcc mutants N417S and N417T were not recognized by a set of broadly neutralizing antibodies: MRCT10.v362, hu5B3.v3, mu5B3 and HCV1; this was attributed to the glycan shift from residue N417 to N415. Most of the published reports, which are based on in situ mutagenesis and recombinant proteins, have focused on the role of glycan shifting in the appearance of neutralization-resistant mutants. Our data may also indicate that HCV E2 glycoproteins with an altered glycosylation pattern, which leads to the possible modulation of E2 conformation, may be less efficient in the stimulation of the host immune response. Moreover, such modulation could also affect the entry function of proteins via the modification of their affinity to HCV receptors. The CD81 receptor plays a key role in the process of HCV entry and the inhibition of the interaction of E1E2 with CD81, which leads to virus neutralization in vitro [26,27]. A conserved Gly436-Tyr443 E2 motif is a determinant of CD81 binding and viral entry [28].

Therefore, we further examined the interaction between CD81 and E2 variants that lack specific N-glycans. The results showed a significant reduction in the CD81 binding of E2 variants. It is worth noting that the variants with the lowest level of CD81 binding, namely NR4 and NR6, both lacked an N3 glycosylation site, which is localized next to the Gly436-Tyr443 E2 motif. Kong and co-workers showed [29] that an additional N-glycosylation site at positions 442 or 428 or a Lys-to-Tyr mutation at position 427 fully inhibited CD81 binding. The collected data indicate that alterations in the E2 N-glycosylation pattern have a major impact on the binding of E2 to CD81. This may be the result of changes in the protein conformation and not necessarily the changes leading to the disruption of the CD81 receptor binding site. Structural flexibility was observed in several E2 domains, including the region containing the glycosylation sites N1, N2, N6, N7, and N10 [30]. Khera et al. [31] reported that the mutations in E2 causing the disruption of N-glycosylation sites led to the lower recognition of CD81 receptors. However, in this report, the inactivation of glycosylation site 534 was accompanied by the deletion of HVR1 and the removal of this region seemed to be the key factor changing the binding to CD81. Nevertheless, in IFN-non-responding patients with a persistent viral infection, E2 affinity to the CD81 receptor may become less important in favor of direct virus cell-to-cell transfer. HCV direct transmission, which is CD81-independent, helps the virus to avoid antibody neutralization and become more persistent [32].

To further analyze the possible changes in the conformation of the tested E2 variants, we evaluated them via an ELISA assay using a panel of conformation-sensitive neutralizing antibodies. The E2 variants obtained from the NR patients were less efficiently recognized by four conformational-sensitive antibodies (HC-1, HC-11, CBH-4B, HC84.1), indicating the presumably altered local conformation of the E2 glycoprotein. The studies conducted by Prentoe and co-authors [33], which are based on cell culture infectious HCV, demonstrated that the effects of changes in the glycosylation pattern were variable. They showed that the removal of glycans at some positions (e.g., N1) makes the virus more sensitive to neutralizing antibodies, while such an effect was not observed for sites N5 or N9. Moreover, the removal of other glycosylation sites (e.g., N3 and N9) was associated with a decreased infectivity in HCVcc. These studies and the work of other authors [34] have indicated that the role of glycans in HCV infection cannot be explained only by simple direct steric epitope shielding, but also by their indirect influence on the dynamic conformational changes that occur in the E2 protein. Some such changes may be genotype-specific, as the authors showed differences in the impact of the removal of individual glycosylation sites in J6 (gt 2a), H77 (gt1a), and gt3a HCVcc.

The results presented in this paper may suggest that alterations in the N-glycosylation pattern of the HCV E2 glycoprotein present at the beginning of anti-HCV treatment may be associated with a negative outcome of therapy. The clearance of the HCV infection depends upon the efficient activity of both the humoral and cellular immune response. It can be hypothesized that the pool of antibodies that is produced during infection with HCV mutants characterized in this report did not provide sufficient protection against the virus; consequently, the antiviral effect of the interferon/ribavirin treatment could have been weakened. The resistance of HCV to interferon is mediated mostly by the ISIDR (interferon sensitivity determination) of NS5A; however, the region of E2, which interacts with protein kinase R (PKR), was also indicated to be associated with resistance to IFN. The PePHD region of E2 (PKR/eIF-2a phosphorylation homology domain), which is located in the C-terminal part of E2 (amino acid 659 to 670), was found to be involved in response to IFN. However, several studies have not confirmed any significant correlations between mutations in the PePHD and patients’ response to IFN therapy [35]. Our analysis was focused on the E2 regions containing glycosylation sites and the most conserved epitopes.

Glycosylation of viral proteins can also affect viral infectivity. To analyze this matter, we have generated HCV pseudo-particles (HCVpp) carrying patient-derived E2 glycoprotein variants and used them for the infectivity studies but the results were inconclusive. However, it has been previously described that mutations leading to the loss of E1N1, E1N4, E2N8, or E2N10 may influence the E1E2 glycoproteins incorporation into HCVpp presumably due to incorrect protein folding. Moreover, changes in E2N2 and E2N4 decreased HCVpp infectivity even though glycoprotein incorporation into HCVpp was not affected [36]. The results of another experiment using HCV pseudo-particles in insect and mammalian cells showed that the removal of glycans present at the E2N1 and E2N8 glycosylation sites has led to the accumulation of E1E2 aggregates and the suppression of virions assembly. Those misfolded E1E2 aggregates were not able to interact with calnexin and, eventually, form the functional glycoprotein complex [37].

Numerous reports have revealed the impact of glycan structures on the virus infectious cycle, as well as on the host immune response and the mechanism implicated in immune escape. The most known examples of extensive studies on the influence of N-glycans on the function of proteins involve influenza hemagglutinin [38], HIV env proteins [39], and, more recently, the SARS-CoV-2 S protein [40,41]. In many of these studies, viral glycosylation has been investigated both as a potential therapy target and in the context of vaccine design. The huge volume of data concerning the complexity of glycans and the importance of the glycosylation of viral proteins is a new field of research; which is defined as the glycoproteomics of viruses [42]. The majority of data regarding the effect of the removal or addition of viral N-glycans is based on the analysis of recombinant proteins generated in vitro meanwhile, there are relatively few glycoproteomic studies of viral proteins derived directly from patients. The findings presented in this report were obtained via an analysis of HCV sequences isolated from clinical samples. Therefore, our observations may contribute to the understanding of the role of viral N-glycosylation in the context of HCV infection. Although we observed a correlation between aberrant glycosylation and lack of response to interferon-ribavirin therapy, the number of samples was too limited to conclude that the lack of glycans is directly related to clinical observations.

Further studies of the dynamic glycosylation changes of viral proteins in HCV clinical isolates are needed to determine the impact of glycosylation of viral proteins on the efficacy of virus infection. The application of new glycosylation research technologies for glycan structure studies can facilitate the development of virus vaccines and antiviral drugs.

## Figures and Tables

**Table 1:** Baseline characteristics of children patients with chronic hepatitis C before antiviral treatment and the therapy outcome. Figlerowicz et al., 2010 [11].

Sample No.	Patient No.	Therapy Outcome	Age (years)	Sex	Pretreatment Viremia (copies/mL)	Pretreatment ALT (IU/L)	Pretreatment Anti-HCV Antibodies
SR1	P2-17	Sustained Response	8	Male	56,300,000	19	+
SR2	P2-18	Sustained Response	15	Female	696,000	54	+
SR3	P2-20	Sustained Response	15	Female	152,000	37	+
TR	P2-24	Transient Response	16	Female	286,000	28	+
NR1	P2-10	No Response	11	Male	2,670,000	45	-
NR2	P2-23	No Response	10	Male	840,000	63	+
NR3	P2-28	No Response	14	Female	365,000	51	-
NR4	P2-05	No Response	11	Female	2,170,000	53	-
NR5	P2-19	No Response	10	Female	726,000	35	+
NR6	P2-04	No Response	13	Male	11,280,000	315	+

**Table 2:** The list of mutations in a consensus Asn-X-Ser/Thr sequence observed in NR-derived E2 sequences. NR—no response, N1-N9—N-glycosylation sites.

Sample	Lacking N-glycosylation Site (Amino Acid No.)	Wild-type N-glycosylation Site Consensus Sequence	Mutation in N-glycosylation Site Sequence
NR1	N1 (AA417)	Asn-Gly-Ser	Asn-Gly-Asn
NR2	N5 (AA476)	Asn-Gly-Ser	Asn-Gly-Arg
	N8 (AA556)	Ans-Ser-Thr	Ser-Ser-Thr
NR3	N1 (AA417)	Asn-Gly-Ser	Asn-Gly-Asn
	N5 (AA476)	Asn-Gly-Ser	Asp-Gly-Ser
NR4	N1 (AA417)	Asn-Gly-Ser	Asn-Gly-Arg
	N3 (AA430)	Asn-Asp-Ser	Asp-Asp-Ser
NR5	N9 (AA576)	Asn-Asn-Thr	Asn-Asn-Ala
NR6	N3 (AA430)	Asn-Asp-Ser	Asp-Asp-Ser

## Data Availability

The raw data supporting the conclusions of this article will be made available by the authors on request.

## References

[1] Hepatitis C. https://www.who.int/news-room/fact-sheets/detail/hepatitis-c.

[2] Kenfack-Momo R., Ngounoue M.D., Kenmoe S., Takuissu G.R., Ebogo-Belobo J.T., Kengne-Ndé C., Mbaga D.S., Zeuko’o Menkem E., Lontuo Fogang R., Tchatchouang S. (2024). Global epidemiology of hepatitis C virus in dialysis patients: A systematic review and meta-analysis. PLoS ONE.

[3] Marshall A.D., Willing A.R., Kairouz A., Cunningham E.B., Wheeler A., O’Brien N., Perera V., Ward J.W., Hiebert L., Degenhardt L. (2024). Global HCV and HIV Treatment Restrictions Group. Direct-acting antiviral therapies for hepatitis C infection: Global registration, reimbursement, and restrictions. Lancet Gastroenterol. Hepatol..

[4] Moustafa R., Dubuisson J., Lavie M. (2019). Function of the HCV E1 envelope glycoprotein in viral entry and assembly. Future Virol..

[5] Pantua H., Diao J., Ultsch M., Hazen M., Mathieu M., McCutcheon K., Takeda K., Date S., Cheung T.K., Phung Q. (2013). Glycan shifting on hepatitis C virus (HCV) E2 glycoprotein is a mechanism for escape from broadly neutralizing antibodies. J. Mol. Biol..

[6] Anjum S., Wahid A., Afzal M.S., Albecka A., Alsaleh K., Ahmad T., Baumert T.F., Wychowski C., Qadri I., Penin F. (2013). Additional glycosylation within a specific hypervariable region of subtype 3a of hepatitis C virus protects against virus neutralization. J. Infect. Dis..

[7] LeBlanc E.V., Kim Y., Capicciotti C.J., Colpitts C.C. (2021). Hepatitis C Virus Glycan-Dependent Interactions and the Potential for Novel Preventative Strategies. Pathogens.

[8] Li D., von Schaewen M., Wang X., Tao W., Zhang Y., Li L., Heller B., Hrebikova G., Deng Q., Ploss A. (2016). Altered Glycosylation Patterns Increase Immunogenicity of a Subunit Hepatitis C Virus Vaccine, Inducing Neutralizing Antibodies Which Confer Protection in Mice. J. Virol..

[9] Czarnota A., Offersgaard A., Owsianka A., Alzua G.P., Bukh J., Gottwein J.M., Patel A.H., Bieńkowska-Szewczyk K., Grzyb K. (2023). Effect of Glycan Shift on Antibodies against Hepatitis C Virus E2 412–425 Epitope Elicited by Chimeric sHBsAg-Based Virus-Like Particles. Microbiol. Spectr..

[10] Figlerowicz M., Sluzewski W., Kowala-Piaskowska A., Mozer-Lisewska I. (2004). Interferon alpha and ribavirin in the treatment of children with chronic hepatitis C. Eur. J. Pediatr..

[11] Figlerowicz M., Jackowiak P., Formanowicz P., Kędziora P., Alejska M., Malinowska N., Błażewicz J., Figlerowicz M. (2010). Hepatitis C virus quasispecies in chronically infected children subjected to interferon-ribavirin therapy. Arch. Virol..

[12] Jackowiak P., Kowala-Piaskowska A., Figlerowicz M., Alejska M., Malinowska N., Figlerowicz M. (2012). Evolution of hepatitis C virus hypervariable region 1 in chronically infected children. Virus. Res..

[13] Owsianka A., Tarr A.W., Juttla V.S., Lavillette D., Bartosch B., Cosset F.L., Ball J.K., Patel A.H. (2005). Monoclonal antibody AP33 defines a broadly neutralizing epitope on the hepatitis C virus E2 envelope glycoprotein. J. Virol..

[14] Clayton R.F., Owsianka A., Aitken J., Graham S., Bhella D., Patel A.H. (2002). Analysis of antigenicity and topology of E2 glycoprotein present on recombinant hepatitis C virus-like particles. J. Virol..

[15] Hadlock K.G., Lanford R.E., Perkins S., Rowe J., Yang Q., Levy S., Pileri P., Abrignani S., Foung S.K. (2000). Human monoclonal antibodies that inhibit binding of hepatitis C virus E2 protein to CD81 and recognize conserved conformational epitopes. J. Virol..

[16] Keck Z.Y., Op De Beeck A., Hadlock K.G., Xia J., Li T.K., Dubuisson J., Foung S.K. (2004). Hepatitis C virus E2 has three immunogenic domains containing conformational epitopes with distinct properties and biological functions. J. Virol..

[17] Keck Z.Y., Xia J., Wang Y., Wang W., Krey T., Prentoe J., Carlsen T., Li A.Y., Patel A.H., Lemon S.M. (2012). Human monoclonal antibodies to a novel cluster of conformational epitopes on HCV E2 with resistance to neutralization escape in a genotype 2a isolate. PLoS Pathog..

[18] Yanagi M., Purcell R.H., Emerson S.U., Bukh J. (1997). Transcripts from a single full-length cDNA clone of hepatitis C virus are infectious when directly transfected into the liver of a chimpanzee. Proc. Natl. Acad. Sci. USA.

[19] Bailly C., Thuru X. (2023). Targeting of Tetraspanin CD81 with Monoclonal Antibodies and Small Molecules to Combat Cancers and Viral Diseases. Cancers.

[20] Messina J.P., Humphreys I., Flaxman A., Brown A., Cooke G.S., Pybus O.G., Barnes E. (2015). Global distribution and prevalence of hepatitis C virus genotypes. Hepatology.

[21] Goffard A., Callens N., Bartosch B., Wychowski C., Cosset F.L., Montpellier C., Dubuisson J. (2005). Role of N-linked glycans in the functions of hepatitis C virus envelope glycoproteins. J. Virol..

[22] Deleersnyder V., Pillez A., Wychowski C., Blight K., Xu J., Hahn Y.S., Rice C.M., Dubuisson J. (1997). Formation of native hepatitis C virus glycoprotein complexes. J. Virol..

[23] Dubuisson J., Hsu H.H., Cheung R.C., Greenberg H.B., Russell D.G., Rice C.M. (1994). Formation and intracellular localization of hepatitis C virus envelope glycoprotein complexes expressed by recombinant vaccinia and Sindbis viruses. J. Virol..

[24] Lavie M., Hanoulle X., Dubuisson J. (2018). Glycan Shielding and Modulation of Hepatitis C Virus Neutralizing Antibodies. Front. Immunol..

[25] Dhillon S., Witteveldt J., Gatherer D., Owsianka A.M., Zeisel M.B., Zahid M.N., Rychłowska M., Foung S.K., Baumert T.F., Angus A.G. (2010). Mutations within a conserved region of the hepatitis C virus E2 glycoprotein that influence virus-receptor interactions and sensitivity to neutralizing antibodies. J. Virol..

[26] Pileri P., Uematsu Y., Campagnoli S., Galli G., Falugi F., Petracca R., Weiner A.J., Houghton M., Rosa D., Grandi G. (1998). Binding of hepatitis C virus to CD81. Science.

[27] Fénéant L., Levy S., Cocquerel L. (2014). CD81 and hepatitis C virus (HCV) infection. Viruses.

[28] Drummer H.E., Boo I., Maerz A.L., Poumbourios P. (2006). A conserved Gly436-Trp-Leu-Ala-Gly-Leu-Phe-Tyr motif in hepatitis C virus glycoprotein E2 is a determinant of CD81 binding and viral entry. J. Virol..

[29] Kong L., Giang E., Nieusma T., Kadam R.U., Cogburn K.E., Hua Y., Dai X., Stanfield R.L., Burton D.R., Ward A.B. (2013). Hepatitis C virus E2 envelope glycoprotein core structure. Science.

[30] Ströh L.J., Krey T. (2023). Structural insights into hepatitis C virus neutralization. Curr. Opin. Virol..

[31] Khera T., Behrendt P., Bankwitz D., Brown R.J.P., Todt D., Doepke M., Khan A.G., Schulze K., Law J., Logan M. (2019). Functional and immunogenic characterization of diverse HCV glycoprotein E2 variants. J. Hepatol..

[32] Zeisel M.B., Barth H., Schuster C., Baumert T.F. (2009). Hepatitis C virus entry: Molecular mechanisms and targets for antiviral therapy. Front. Biosci..

[33] Prentoe J., Velázquez-Moctezuma R., Augestad E.H., Galli A., Wang R., Law M., Alter H., Bukh J. (2019). Hypervariable region 1 and N-linked glycans of hepatitis C regulate virion neutralization by modulating envelope conformations. Proc. Natl. Acad. Sci. USA.

[34] Pierce B.G., Keck Z.Y., Lau P., Fauvelle C., Gowthaman R., Baumert T.F., Fuerst T.R., Mariuzza R.A., Foung S.K.H. (2016). Global mapping of antibody recognition of the hepatitis C virus E2 glycoprotein: Implications for vaccine design. Proc. Natl. Acad. Sci. USA.

[35] Muñoz de Rueda P., Casado J., Patón R., Quintero D., Palacios A., Gila A., Quiles R., León J., Ruiz-Extremera A., Salmerón J. (2008). Mutations in E2-PePHD, NS5A-PKRBD, NS5A-ISDR, and NS5A-V3 of hepatitis C virus genotype 1 and their relationships to pegylated interferon-ribavirin treatment responses. J. Virol..

[36] Helle F., Vieyres G., Elkrief L., Popescu C.I., Wychowski C., Descamps V., Castelain S., Roingeard P., Duverlie G., Dubuisson J. (2010). Role of N-linked glycans in the functions of hepatitis C virus envelope proteins incorporated into infectious virions. J. Virol..

[37] Orlova O.V., Drutsa V.L., Spirin P.V., Prasolov V.S., Rubtsov P.M., Kochetkov S.N., Beljelarskaya S.N. (2015). The role of HCV e2 protein glycosylation in functioning of virus envelope proteins in insect and Mammalian cells. Acta Naturae.

[38] Altman M.O., Angel M., Košík I., Trovão N.S., Zost S.J., Gibbs J.S., Casalino L., Amaro R.E., Hensley S.E., Nelson M.I. (2019). Human Influenza A Virus Hemagglutinin Glycan Evolution Follows a Temporal Pattern to a Glycan Limit. mBio.

[39] Jan M., Upadhyay C., Alcami Pertejo J., Hioe C.E., Arora S.K. (2018). Heterogeneity in glycan composition on the surface of HIV-1 envelope determines virus sensitivity to lectins. PLoS ONE.

[40] Newby M.L., Allen J.D., Crispin M. (2023). Influence of glycosylation on the immunogenicity and antigenicity of viral immunogens. Biotechnol. Adv..

[41] Baboo S., Diedrich J.K., Torres J.L., Copps J., Singh B., Garrett P.T., Ward A.B., Paulson J.C., Yates J.R. (2023). Evolving spike-protein N-glycosylation in SARS-CoV-2 variants. bioRxiv.

[42] Bagdonaite I., Wandall H.H. (2018). Global aspects of viral glycosylation. Glycobiology.

